# Genetic variations of *DICKKOPF* family genes might not be associated with gastric cancer susceptibility

**DOI:** 10.1186/s12876-016-0489-1

**Published:** 2016-07-26

**Authors:** Juan Wu, Junfeng Zhang, Zhen Zhan, Qinhong Cao, Zhong Li

**Affiliations:** 1 Department of Nutrition and Food Hygiene, Key Laboratory of Modern Toxicology, Ministry of Education, School of Public Health, Nanjing Medical University, 818 East Tianyuan Rd, Nanjing, China; 2 Discipline of Chinese and Western Integrative Medicine, Nanjing University of Chinese Medicine, Nanjing, China; 3 Department of Digestive Tumor Surgery, Jiangsu Province Hospital of Traditional Chinese Medicine, Affiliated Hospital of Nanjing University of Traditional Chinese Medicine, Nanjing, China

**Keywords:** Wnt signaling pathway, Stomach neoplasms, Single nucleotide polymorphism, Epidemiology

## Abstract

**Background:**

Recent studies have implicated that members of the *DICKKOPF* (*DKK*) were causally involved in large number of human cancers. This study was designed to investigate the relationship between the genetic variations of *DKK* family genes and the risk of gastric cancer (GC).

**Methods:**

Six SNPs (single nucleotide polymorphisms) of *DKK* family genes, including rs2241529 in *DKK1*, rs3733635, rs17037102 and rs419764 in *DKK2*, rs3206824 in *DKK3* and rs2073664 in *DKK4*, were selected and genotyped by restriction fragment length polymorphism (RFLP) and TaqMan SNP genotyping methods in 409 GC cases and 554 cancer-free controls in the Han population in eastern China.

**Results:**

None of the six SNPs achieved significant association with the overall GC risk and stratified analysis by age, gender, smoking status, drinking status, tumor location and pathological classification confirmed these non-significant associations.

**Conclusions:**

Our study indicated that the studied six SNPs of *DKKs* would not be the risk factors for GC in this Han Chinese population. Studies of larger population for different ethnicities will be needed to warrant our findings.

## Background

Based on GLOBOCAN estimates, about 951,600 new gastric cancer (GC) cases and 723,100 deaths occurred in 2012 worldwide [1]. In China, GC rate is predominately high and GC becomes the third fatal causes of caner, following lung cancer and liver cancer [2], which induces enormous burden on the society. The mechanism of gastric carcinogenesis is complex and it is well documented that environmental elements, including *Helicobacter pylori* (*HP*) infection and life styles, such as diet pattern, alcohol consumption and tobacco smoking, may contribute to the predisposition of GC [3]. Meanwhile, some other factors may play an important role in GC etiology, such as epigenetic and genetic effects [4]. Recently, associations between genetic variants and GC risk have been widely investigated [5, 6]. However, the results were not always consistent. Up to date, genetic factors for GC pathogenesis are still not fully interpreted.

As is well known, deregulation of Wnt/β-catenin pathway is a hallmark of major gastrointestinal cancers, including colorectal cancer and GC [7]. Several agonists and antagonists could modulate the Wnt/β-catenin pathway and were involved in the development and progression of malignant tumors. As a member of agonists and antagonists, the Dickkopfs (DKKs) were identified as a group of secreted Wnt modulators. The vertebrates express four DKK proteins (DKK1, -2, -3, and -4) [8]. The cysteine rich secreted protein products of the *DKKs* are antagonists of Wnt glycoproteins through binding to lipoprotein receptor-related protein 5/6 (LRP5/6) and Kremen, thus inducing LRP endocytosis and preventing signaling to β-catenin [9].

Several recent studies have implicated that members of the *DKKs* were causally involved in large number of human cancers including colorectal [10], melanoma [11], hepatocellular carcinoma [12], and GC [13]. In clinical GC specimens, *DKK1* mRNA and protein expression levels were both reported to be significantly upregulated in GC lesions compared to adjacent noncancerous tissues [13]. Meanwhile, it would be a predictor of poor prognosis for GC patients [14].

The genetic variants of *DKK* family genes influencing the DKKs expressions on GC was not well elucidated. We hypothesized that variations of the corresponding *DKKs*, especially for those variants located in the gene functional region, may influence the mRNA and protein expression levels, and thus the predisposition of the disease. Therefore, we conducted a hospital-based case-control study to investigate the association between six potentially functional variants in *DKKs* and GC risk with 409 GC patients and 554 cancer-free healthy controls in a Chinese population.

## Methods

### Study subjects

In this study, 409 GC patients and 554 cancer-free healthy controls were recruited from the Jiangsu Provincial Hospital of Traditional Chinese Medicine (TCM) from January 2008 to July 2012. The patients were histo-pathologically confirmed primary GC. GC cases were classified into intestinal and diffuse subtype according to the Lauren’s criteria and those with mixed type or not available for classification were denoted unclassified. Gastric cardia cancers were defined as tumors located within 20 mm distal to the gastro-esophageal junction. The healthy controls were selected from the visitors of the health examination clinic of Jiangsu Provincial Hospital of TCM who came for an annual check-up during the same study period. The inclusion and exclusion criteria of the control subjects referred to the previous study [15]. Peripheral blood samples were obtained from the GC cases and healthy controls after the written informed consent was received from each subject. The study was approved by the Institutional Review Board of Jiangsu Provincial Hospital of TCM.

### Genotyping of DKKs polymorphisms

Genomic DNA was extracted by AxyPrep-96 kit (Axygen, CA, USA) following the manufacturer’s instructions, and was quantified spectrophotometrically on NanoDrop2000 (Thermo Scientific, USA). From the public SNP database (http://www.ncbi.nlm.nih.gov/snp), 6 SNPs of the *DKKs* (rs2241529 in *DKK1*; rs3733635, rs17037102 and rs419764 in *DKK2*; rs3206824 in *DKK3* and rs2073664 in *DKK4*) were selected as the candidate SNPs with minor allele frequency more than 10 % in the Han Chinese population. All the selected SNPs were located in the potentially functional region of the genes (5′UTR, exon, and 3′UTR), and the restriction fragment length polymorphism (RFLP) method was applied for genotyping the four SNPs rs3733635, rs17037102, rs3206824, and rs2073664. For those SNPs (SNPs rs419764 and rs2241529) with no proper restriction endonuclease, the commercially available predesigned TaqMan MGB probes and designed primers (Applied Biosystems, Foster City, CA, USA) were used for the genotyping. PCR reaction was conducted on the Veriti® 96-Well Thermal Cycler and the QuantStudio™ Dx Real-Time PCR Instrument (Applied Biosystems, Foster City, CA, USA), and the reaction condition can be referred to the previous study [16]. All the primers, enzymes and TaqMan SNP genotyping assay IDs are shown in Table 2.

### Statistical analyses

The two sided χ^2^ test was applied to compare the genotype frequency of the *DKKs* variants between the GC cases and the controls. For the association analysis, the logistic regression analyses were employed to estimate the association between *DKKs* genotypes and risk of GC by computing the odds ratios (ORs) and 95 % confidence intervals (CIs), all the ORs were adjusted by age (continuous value) and sex (male = 0, female = 1). The common homozygote was selected as the reference for calculating the genotype specific ORs. Bonferroni correction was applied for multiple comparison. The Hardy-Weinberg equilibrium was tested by the χ^2^ test for goodness-of-fit and the *p*-value less than 0.05 was considered as the significant level. All the analyses were performed by the SAS 9.1.3 software (SAS Institute, Inc., Cary, NC, USA).

## Results

Four hundred nine GC cases and 554 cancer-free healthy controls were enrolled in this study. The mean age for the cases and controls were 59.9 ± 11.2 and 53.5 ± 13.4 years, respectively. The proportion of male subjects was 70.9 % among the GC cases, and 64.4 % among the controls. Smokers accounted of 40.1 % among the GC cases, and 31.0 % among the healthy controls. Drinkers were 23.5 % among the GC cases, and 15.0 % in the healthy controls. Of the 409 GC cases, 292 (71.4 %) patients were classified as non-cardia GC and 106 (25.9 %) patients were classified as cardia GC according the tumor location. Meanwhile, 11 (2.7 %) cases cannot provide tumor location for the classification. For the GC cases, 383 (94.3 %) pathological tissues of each patient were available for the histological type dividing (72 diffuse GC cases and 311 intestinal GC cases) according to the Lauren’s classification. However, 26 (6.4 %) patients’ pathological tissues were not available for the classification (Table 1).

**Table 1:** Distributions of basic characteristics of GC cases and controls

Variables	Controls *n* = 554 (%)	Cases *n* = 409 (%)	*P*
Age	53.5 ± 13.4	59.9 ± 11.2	<0.001
Gender			0.035
Male	357 (64.4)	290 (70.9)	
Female	197 (35.6)	119 (29.1)	
Smoke			0.004
No	382 (69.0)	245 (59.9)	
Yes	172 (31.0)	164 (40.1)	
Drink			0.008
No	471 (85.0)	313 (76.5)	
Yes	83 (15.0)	96 (23.5)	
Tumor site			
Cadia		106 (25.9)	
Non-cardia		292 (71.4)	
Unclear		11 (2.7)	
Pathological type			
Intestinal		311 (76.6)	
Diffuse		72 (17.7)	
Unclear		26 (6.4)	

The genotype distribution of the six SNPs of *DKKs* among the GC cases and the controls is shown in Table 3. The observed genotype frequencies for all the SNPs were in Hardy-Weinberg equilibrium among the controls (*P* > 0.05) (Table 2). Allele frequencies of all the six SNPs (rs2241529, rs3733635, 17037102, 419764, 3206824 and rs2073664) among the GC and the controls demonstrated no significant difference (*P* = 0.42, 0.30, 0.81, 0.25, 0.99 and 0.94, respectively). None of the six SNPs achieved significant differences for the genotype distribution between GC cases and controls (*P* = 0.67 for rs2241529, 0.44 for rs3733635, 0.94 for 17037102, 0.28 for 419764, 0.90 for3206824 and 0.64 for rs2073664, respectively). Multiple logistic regression analysis revealed that none of the six SNPs in *DKKs* were associated with the overall GC risk (all *P* values > 0.05) (Table 3). The genetic effects of the six loci were further evaluated on the GC risk according to the confirmed histological type (intestinal and diffuse type), and the GA genotype of rs17037102 in *DKK2* was found in association with an increased risk of diffuse GC with only marginal significance (adjusted OR = 1.77, 95 % CI: 1.01-3.11, *P* = 0.05). However, after Bonferroni correction, the *P* value reached no statistical significance (*P* = 0.14). GA genotype of rs17037102 was also not found in association with the intestinal GC (*P* = 0.42) (Table 4). Meanwhile, according to the site of tumor origin, it was not found any SNP was in association with the risk of either gastric non-cardia cancer or gastric cardia cancer (all *P* values >0.05) (Table 3).

**Table 2:** The genotyping methods for the SNPs of *DKK* family genes and Hardy-Weinberg equilibrium for control group

SNP ID	Gene	Assay method	Primers		Enzymes	PCR product Length	Alleles	Genotype and corresponding fragments length	Assay_ID	P*
rs2241529	*DKK1*	TaqMan	NA	NA	NA	NA	A > G	NA	C__15873446_20	0.28
rs419764	*DKK2*	TagMan	NA	NA	NA	NA	C > T	NA	C___2543897_10	0.23
rs3733635	*DKK2*	RFLP	Forward	ATTTCTGTCCTGAGGCGTGA	HinfI	170	T > C	TT (170) TC (170, 145, 25) CC (145,25)	NA	0.39
			Reverse	TCCCCAGGAAGACGCAAAG					NA	
rs17037102	*DKK2*	RFLP	Forward	TCTTAACCCCTCACATCCCG	DdeI	151	G > A	GG (117, 34) GA (117, 75, 42, 34) AA (75, 42, 34)	NA	0.88
			Reverse	CCAGAACTACAGATATCCCTACC					NA	
rs3206824	*DKK3*	RFLP	Forward	GGAGAGGAGCCTGACTGAAG	DdeI	232	G > A	242 (GG) GA (242, 203, 29) AA (203, 29)	NA	0.44
			Reverse	ATGCACAACACCTCATGCTG					NA	
rs2073664	*DKK4*	RFLP	Forward	GGTCCCATTCCCTTATCCCA	EcoNI	248	C > T	CC (181, 67) CT (248, 181, 67) TT (248)	NA	0.66
			Reverse	CGCTGGAAGATTTCTGGAGC					NA	

Abbreviations: *NA* not applicable

*P value for Hardy-Weinberg equilibrium of the studied genotypes in the controls

**Table 3:** The relationship between SNPs of *DKK* family genes and GC and subtype GC by tumor site

Genotypes	Controls (*N* = 554)		Cases (*N* = 409)		Adjusted OR (95 % CI)^a^			*P*	Cardia (*N* = 106)		Adjusted OR (95 % CI)^a^			*P*	Non-Cardia (*N* = 292)		Adjusted OR (95 % CI)^a^			*P*
	n	%	n	%					n	%					n	%				
rs2241529(*DKK1*)																				
AA	260	46.9 %	184	45.0 %	1.00				42	39.6 %	1.00				136	46.6 %	1.00			
AG	247	44.6 %	184	45.0 %	1.07	0.81	1.41	0.64	52	49.1 %	1.35	0.86	2.14	0.20	127	43.5 %	1.01	0.75	1.37	0.94
GG	47	8.5 %	41	10.0 %	1.31	0.82	2.10	0.27	12	11.3 %	1.59	0.76	3.34	0.22	29	9.9 %	1.25	0.74	2.09	0.40
AG + GG/AA	294	53.1 %	225	55.0 %	1.11	0.85	1.44	0.46	64	60.4 %	1.39	0.90	2.16	0.14	156	53.4 %	1.05	0.79	1.40	0.75
rs3733635(*DKK2*)																				
TT	335	60.5 %	238	58.2 %	1.00				61	57.5 %	1.00				169	57.9 %	1.00			
TC	196	35.4 %	147	35.9 %	1.11	0.84	1.47	0.46	40	37.7 %	1.26	0.80	1.98	0.32	105	36.0 %	1.11	0.82	1.51	0.49
CC	23	4.2 %	24	5.9 %	1.40	0.76	2.59	0.28	5	4.7 %	1.40	0.49	4.02	0.53	18	6.2 %	1.45	0.75	2.79	0.27
TC + CC/AA	219	39.5 %	171	41.8 %	1.14	0.88	1.49	0.33	45	42.5 %	1.28	0.82	1.98	0.28	123	42.1 %	1.15	0.86	1.54	0.35
rs17037102(*DKK2*)																				
GG	214	38.6 %	157	38.4 %	1.00				39	36.8 %	1.00				114	39.0 %	1.00			
GA	262	47.3 %	191	46.7 %	0.98	0.74	1.31	0.91	50	47.2 %	1.04	0.65	1.67	0.87	135	46.2 %	0.96	0.70	1.31	0.78
AA	78	14.1 %	61	14.9 %	1.08	0.72	1.62	0.70	17	16.0 %	1.30	0.68	2.50	0.42	43	14.7 %	1.04	0.67	1.62	0.85
GA + AA/GG	340	61.4 %	252	61.6 %	1.01	0.77	1.32	0.97	67	63.2 %	1.10	0.70	1.71	0.69	178	61.0 %	0.98	0.73	1.31	0.88
rs419764(*DKK2*)																				
CC	304	54.9 %	244	59.7 %	1.00				61	57.5 %	1.00				176	60.3 %	1.00			
CT	220	39.7 %	142	34.7 %	0.80	0.61	1.06	0.11	38	35.8 %	0.88	0.56	1.39	0.58	100	34.2 %	0.79	0.58	1.07	0.13
TT	30	5.4 %	23	5.6 %	0.94	0.52	1.68	0.83	7	6.6 %	1.10	0.45	2.71	0.83	16	5.5 %	0.89	0.47	1.70	0.72
CT + TT	250	45.1 %	165	40.3 %	0.82	0.63	1.06	0.13	45	42.5 %	0.91	0.59	1.40	0.66	116	39.7 %	0.80	0.60	1.08	0.14
rs3206824(*DKK3*)																				
GG	340	61.4 %	249	60.9 %	1.00				60	56.6 %	1.00				182	62.3 %	1.00			
GA	184	33.2 %	140	34.2 %	1.06	0.80	1.40	0.69	41	38.7 %	1.34	0.85	2.11	0.20	96	32.9 %	0.98	0.72	1.34	0.92
AA	30	5.4 %	20	4.9 %	0.94	0.51	1.72	0.84	5	4.7 %	0.87	0.31	2.38	0.78	14	4.8 %	0.89	0.46	1.74	0.73
GA + AA/GG	214	38.6 %	160	39.1 %	1.04	0.80	1.36	0.76	46	43.4 %	1.27	0.82	1.96	0.29	110	37.7 %	0.97	0.72	1.31	0.85
rs2073664(*DKK4*)																				
CC	444	80.1 %	331	80.9 %	1.00				85	80.2 %	1.00				236	80.8 %	1.00			
CT	105	19.0 %	72	17.6 %	0.91	0.64	1.27	0.56	18	17.0 %	0.82	0.46	1.44	0.49	53	18.2 %	0.93	0.64	1.35	0.71
TT	5	0.9 %	6	1.5 %	1.39	0.41	4.74	0.60	3	2.8 %	2.92	0.62	13.73	0.17	3	1.0 %	0.91	0.21	3.89	0.89
CT + TT/CC	110	19.9 %	78	19.1 %	0.93	0.67	1.29	0.66	21	19.8 %	0.91	0.53	1.56	0.73	56	19.2 %	0.93	0.65	1.34	0.70

^a^ORs were adjusted by age and gender

**Table 4:** The association between SNPs of *DKK* family genes and the pathological subtype GC cases

Genotypes	Controls (*N* = 554)		Intestinal (*N* = 311)		Adjusted OR (95 % CI)^a^			*P*	Diffuse (*N* = 72)		Adjusted OR (95 % CI)^a^			*P*
	n	%	n	%					n	%				
rs2241529(DKK1)														
AA	260	46.9 %	141	45.3 %	1.00				29	40.3 %	1.00			
AG	247	44.6 %	142	45.7 %	1.10	0.82	1.49	0.52	34	47.2 %	1.25	0.73	2.11	0.42
GG	47	8.5 %	28	9.0 %	1.10	0.65	1.87	0.72	9	12.5 %	1.75	0.78	3.96	0.18
AG + GG/AA	294	53.1 %	170	54.7 %	1.10	0.83	1.47	0.51	43	59.7 %	1.33	0.80	2.19	0.27
rs3733635(DKK2)														
TT	335	60.5 %	190	61.1 %	1.00				38	52.8 %	1.00			
TC	196	35.4 %	100	32.2 %	0.98	0.72	1.34	0.91	32	44.4 %	1.48	0.89	2.45	0.13
CC	23	4.2 %	21	6.8 %	1.58	0.84	3.00	0.16	2	2.8 %	0.77	0.17	3.39	0.73
TC + CC/AA	219	39.5 %	121	38.9 %	1.05	0.78	1.41	0.75	34	47.2 %	1.40	0.85	2.30	0.18
rs17037102(DKK2)														
GG	214	38.6 %	124	39.9 %	1.00				20	27.8 %	1.00			
GA	262	47.3 %	139	44.7 %	0.88	0.64	1.20	0.42	43	59.7 %	1.77	1.01	3.11	0.05*
AA	78	14.1 %	48	15.4 %	1.08	0.70	1.66	0.74	9	12.5 %	1.27	0.55	2.92	0.57
GA + AA/GG	340	61.4 %	187	60.1 %	0.92	0.69	1.24	0.59	52	72.2 %	1.66	0.96	2.86	0.07
rs419764(DKK2)														
CC	304	54.9 %	179	57.6 %	1.00				48	66.7 %	1.00			
CT	220	39.7 %	112	36.0 %	0.87	0.64	1.18	0.36	23	31.9 %	0.66	0.39	1.12	0.13
TT	30	5.4 %	20	6.4 %	1.14	0.61	2.10	0.69	1	1.4 %	0.20	0.03	1.48	0.11
CT + TT	250	45.1 %	132	42.4 %	0.90	0.68	1.20	0.48	24	33.3 %	0.60	0.36	1.02	0.06
rs3206824(DKK3)														
GG	340	61.4 %	193	62.1 %	1.00				38	52.8 %	1.00			
GA	184	33.2 %	103	33.1 %	0.99	0.73	1.35	0.97	29	40.3 %	1.43	0.85	2.41	0.17
AA	30	5.4 %	15	4.8 %	0.87	0.45	1.69	0.68	5	6.9 %	1.47	0.54	4.04	0.45
GA + AA/GG	214	38.6 %	118	37.9 %	0.98	0.73	1.31	0.87	34	47.2 %	1.44	0.88	2.36	0.15
rs2073664(DKK4)														
CC	444	80.1 %	252	81.0 %	1.00				60	83.3 %	1.00			
CT	105	19.0 %	54	17.4 %	0.88	0.60	1.28	0.49	11	15.3 %	0.76	0.39	1.50	0.43
TT	5	0.9 %	5	1.6 %	1.55	0.43	5.62	0.50	1	1.4 %	1.30	0.15	11.48	0.81
CT + TT/CC	110	19.9 %	59	19.0 %	0.91	0.63	1.31	0.61	12	16.7 %	0.79	0.41	1.52	0.47

*P value reached no statistical significance by Bonferroni correction

^a^ORs were adjusted by age and gender

## Discussion

In this study, rs2241529 in *DKK1*, rs3733635, rs17037102 and rs419764 in *DKK2*, rs3206824 in *DKK3*, and rs2073664 in *DKK4* were analyzed among 409 pathologically confirmed GC patients and 554 healthy controls. The relationship between these SNPs and clinic pathologic data, including pathological subtype (intestinal and diffuse) and tumor original site (gastric cardia and non-cardia cancer) was analyzed. Our results showed an insignificant association, as well as among a series subgroup analysis and this is the first study to assess these six variants of *DKK* family genes on the risk of GC.

DKKs are known as antagonists of Wnt glycoproteins through binding to lipoprotein LRP5/6 and Kremen, thus inducing LRP endocytosis and preventing the signaling to β-catenin [9]. Human DKKs have been implicated in several kinds of malignant tumors. For instance, DKK1 protein was predominantly elevated in tissues of hepatocellular carcinoma [12], non-small cell lung cancer [17] and chondrosarcoma [18]. For GC, DKK1 protein was also expressed higher in malignancy than benign tissues [14] and could refer to poor prognosis [13]. DKK2 protein was seen overexpressed in Ewing sarcoma [19], colorectal cancer [10] and reduce in melanoma [11]. It may function as a tumor suppressor or tumor activator depending on the circumstance. DKK3 and DKK4 could be referred as the putative tumor suppressors. Tumor suppression role of DKK3 protein was found in human cancers in ovary [20], cervix [21] and colon [22]; and expression of DKK4 protein was reduced in hepatocellular carcinoma tissue and could decrease the β-catenin protein levels [12].

Variation of the corresponding *DKKs*, especially in the potentially functional regions, may influence the mRNA and protein expression, and thus the risk of tumors. A small case-control study (210 renal patients vs. 200 controls) reported GA/AA genotype of rs3206824 in *DKK3* and GG genotype of rs17037102 in *DKK2* were related with decreased risk of renal cancer and cancer deaths, respectively, in Japanese population [23]. However, our study did not reappear such an association in GC risk in Chinese population. The results suggest a considerable heterogeneous effect of these two SNPs among various cancer types and/or different genetic backgrounds. Alanazi et al., [24] reported 2-fold reduced breast cancer risk in women with GG genotype as compared to AA genotype in case of rs6485350 in *DKK3* [24]. Furthermore, GG genotype and AG genotype showed enhanced protection against estrogen receptor positive tumor and estrogen receptor negative tumor, respectively. SNP rs3763511 in *DKK4* was related with age independent increased breast cancer risk of estrogen receptor negative tumor. Despite the significant findings, two loci located in the introns in the corresponding gene and beyond our selection range. The significant association could be explained by the linkage disequilibrium with the potentially functional loci, or the observed association may be due to chance with small sample size (99 patients vs. 93 controls).

Several limitations of the present study need to be mentioned. First, the mean age in the GC cases was significantly higher than that of the healthy controls, and the age itself may be an independent risk element for GC. However, all the odds ratio of six SNPs was adjusted by age and gender to minimize the potential confounding effect. Second, environmental factors, such as *H. pylori* infection was not considered in this study, which may demonstrate the non-genetic factors on the risk of GC, and further explain the gene-environmental interaction on GC. Further studies with large sample size are warranted to evaluate the contribution of gene-environment interaction on GC.

## Conclusions

Our study indicated that the studied six SNPs of *DKKs* would not be the risk factors for GC in this Han Chinese population. However, analysis of these SNPs incorporating with environmental factors may further explain the risk on GC.

## Abbreviations

CI, confidence interval; DKK, DICKKOPF; GC, gastric cancer; LRP5/6, lipoprotein receptor-related protein 5/6; OR, odds ratio; RFLP, restriction fragment length polymorphism; SNP, single nucleotide polymorphism; TCM, traditional Chinese medicine.
